# Theranostic Sorafenib-Loaded Polymeric Nanocarriers Manufactured by Enhanced Gadolinium Conjugation Techniques

**DOI:** 10.3390/pharmaceutics11100489

**Published:** 2019-09-23

**Authors:** Tivadar Feczkó, Albrecht Piiper, Thomas Pleli, Christian Schmithals, Dominic Denk, Stephanie Hehlgans, Franz Rödel, Thomas J. Vogl, Matthias G. Wacker

**Affiliations:** 1 Research Centre for Natural Sciences, Hungarian Academy of Sciences, Magyar tudosok krt. 2., H-1117 Budapest, Hungary; 2 Research Institute of Biomolecular and Chemical Engineering, University of Pannonia, Egyetem u. 2., H-8200 Veszprém, Hungary; 3 Department of Medicine 1, University Hospital Frankfurt, Theodor-Stern-Kai 7, D-60590 Frankfurt, Germany; Piiper@med.uni-frankfurt.de (A.P.); thomas_pleli@yahoo.com (T.P.); Christian.Schmithals@kgu.de (C.S.); domdenk@googlemail.com (D.D.); 4 Department of Radiotherapy and Oncology, University Hospital Frankfurt am Main, Theodor-Stern-Kai 7, D-60590 Frankfurt am Main, Germany; Stephanie.Hehlgans@kgu.de (S.H.); Franz.Roedel@kgu.de (F.R.); 5 Department of Diagnostic and Interventional Radiology, University Hospital Frankfurt, Theodor-Stern-Kai 7, D-60590 Frankfurt, Germany; Thomas.Vogl@kgu.de; 6 Department of Pharmacy, National University of Singapore, 6 Science Drive 2, Singapore 117546, Singapore; phamgw@nus.edu.sg

**Keywords:** gadolinium, drug release, polymeric nanocarrier, sorafenib, theranostic nanoparticles

## Abstract

Today, efficient delivery of sorafenib to hepatocellular carcinoma remains a challenge for current drug formulation strategies. Incorporating the lipophilic molecule into biocompatible and biodegradable theranostic nanocarriers has great potential for improving the efficacy and safety of cancer therapy. In the present study, three different technologies for the encapsulation of sorafenib into poly(d,l-lactide-*co*-glycolide) and polyethylene glycol-poly(d,l-lactide-*co*-glycolide) copolymers were compared. The particles ranged in size between 220 and 240 nm, with encapsulation efficiencies from 76.1 ± 1.7% to 69.1 ± 10.1%. A remarkable maximum drug load of approximately 9.0% was achieved. Finally, a gadolinium complex was covalently attached to the nanoparticle surface, transforming the nanospheres into theranostic devices, allowing their localization using magnetic resonance imaging. The manufacture of sorafenib-loaded nanoparticles alongside the functionalization of the particle surface with gadolinium complexes resulted in a highly efficacious nanodelivery system which exhibited a strong magnetic resonance imaging signal, optimal stability features, and a sustained release profile.

## 1. Introduction

Liver cancer, of which the majority of cases are hepatocellular carcinoma (HCC), is a life-threatening disease and, according to global cancer statistics, the third leading cause of cancer-related mortality worldwide [1]. To date, sorafenib is the only drug able to prolong the lives of patients with HCC [2], although at the expense of severe side effects due to uptake of the drug into healthy tissues [3]. Sorafenib is a multi-kinase inhibitor targeting various receptor tyrosine kinases and rapidly accelerated fibrosarcoma (RAF) kinases. The pronounced lipophilicity of the molecule is responsible for its poor bioavailability and the distribution of the compound into healthy tissues [3]. As a consequence, patients are treated using high doses of the drug and suffer from a number of side effects.

Nanocarrier-based delivery of sorafenib has the potential to improve drug therapy significantly. Theranostic nanodelivery systems offer a versatile combination of therapeutic and diagnostic features, and have previously been applied to this task [4]. A variety of contrast agents, such as gadolinium diethylenetriamine pentaacetic acid (Gd-DTPA), shorten the longitudinal relaxation time, and have been widely used for both vascular and tumor magnetic resonance imaging (MRI) [4,5]. One major limitation of this technique lies in the short half-life of the contrast agent, as well as its poor specificity for the target site. 

In this context, Gd-DTPA-conjugated human serum albumin (HSA) nanoparticles improved the contrast of MRI compared to free Gd-DTPA aqueous solution in vivo, due to negative contrasting of the tumors [5]. Even after conjugation of polyethylene glycol (PEG) to the particle surface, HSA nanoparticles exhibited only a short circulation time [6] and the scale-up potential of this technology is rather limited [7]. 

Block copolymers comprising polylactic-*co*-glycolic acid (PLGA) and PEG have been processed to nanoparticles in pilot scale using either microfluidic technologies [8] or emulsion techniques [9]. Recently, targeted nanotherapeutic formulations successfully passed phase 1 clinical trials [10]. A major shortcoming of nanocarrier delivery is limited drug loading, resulting in high excipient concentrations and administration volume [11]. 

Several preparation techniques of PLGA nanoparticle preparation have been described in the literature. Previous investigations reported the loading of sorafenib into PLGA nanoparticles, achieving a drug load of 1.4% oil-in-water single emulsion–solvent evaporation method [12]. Preparations made with a nanoprecipitation–dialysis technique using a block copolymer comprising dextran and PLGA resulted in a drug load of 5.3% [13]. To attain synergistic effects of cytostatic agents, co-delivery of drug molecules has been considered. By employing a sequential freeze–thaw method followed by ethanol coacervation, a core–shell construct was manufactured [14]. After preparation of a polyvinyl alcohol (PVA)-doxorubicin nanocore, a thin shell of HSA covering sorafenib was used as a second drug molecule. A drug load of 2.4% was reached. Lipid–polymer hybrid nanoparticles were synthesized for the co-delivery of doxorubicin and sorafenib to enhance efficacy in HCC therapy [15].

Other approaches have focused on theranostic drug delivery systems containing gadolinium (Gd) and sorafenib. Theranostic liposomal carriers with a drug content of 4.3% (*m/m*) have been produced [16]. Another system used a multiblock polymer comprising (poly(lactic acid)-poly(ethylene glycol)-poly(l-lysine)-diethylenetriamine pentaacetic acid and the pH-sensitive material poly(l-histidine)-poly(ethylene glycol)-biotin. A drug content of 2.4% (*m/m*) of sorafenib was reached, and the MRI signal intensity was more beneficial than that of Magnevist^®^ in vivo [12].

In the present study, the impact of three different preparation techniques on the physicochemical features of poly(d,l-lactide-*co*-glycolide) (PLGA) and polyethylene glycol-copolymer (PEG-PLGA) nanocarriers were compared. Nanocarriers loaded with sorafenib were manufactured using the nanoprecipitation, single emulsion, and double emulsion–solvent evaporation methods. Nanoparticles manufactured using the single emulsion–solvent evaporation method were further optimized with regards to the intended target product profile. In vitro cytotoxicity and cellular uptake were investigated in HepG2 cells. Finally, the nanoparticles were modified on their surface using a gadolinium complex, resulting in a theranostic nanocarrier system. For this purpose, the encapsulation of HSA into nanoparticles and the covalent modification of the surface using HSA or polyethylene imine were considered. 

## 2. Materials and Methods

### 2.1. Materials

Resomer^®^ RG 502H (PLGA, lactide: glycolide: 50:50, inherent viscosity: 0.16–0.24 dL/g), Resomer^®^ RG 752H (PLGA, lactide:glycolide: 75:25 inherent viscosity 0.14–0.22 dL/g), and block copolymer Resomer^®^ RGP d5055 (PEG-PLGA, PEG content: 3–7% (*m/m*), inherent viscosity: 0.93 dL/g) were obtained from Evonik Industries AG (Essen, Germany). PVA (*M*_w_ = 30,000–70,000, 87–90% hydrolysed), polysorbate 80, Triton X-100, Pluronic F127, poly(methacrylic acid sodium salt) emulsifiers, dichloromethane (DCM), acetone, dimethyl sulfoxide (DMSO), sodium azide, D-trehalose dehydrate, mannitol, polyethyleneimine (PEI) (MW 25 kDa), 1-Ethyl-3-(3-dimethylaminopropyl)carbodiimide (EDC), *N*-hydroxysuccinimide (NHS), Gd-DTPA, and HSA were obtained from Sigma Aldrich (St. Louis, MO, USA). Sorafenib (free base) was purchased from LC Laboratories (Woburn, MA, USA). Magnevist^®^ was purchased from Bayer AG (Leverkusen, Germany). The micro bicinchoninic acid (µBCA) protein assay kit was bought from Pierce Biotechnology, Inc. (Waltham, MA, USA).

### 2.2. Cell Culture Experiments in HepG2 Cells

The human hepatoma cell line HepG2 was grown in Dulbecco’s modified Eagle’s medium supplemented with 10% fetal calf serum (FCS), 100 U/mL penicillin, and 100 μg/mL streptomycin. The cells were cultured at 37 °C in a humidified atmosphere containing 5% carbon dioxide. The cells were trypsinised, resuspended, and precultured before use.

### 2.3. Preparation of Nanoparticles Using Nanoprecipitation

In brief, 5 to 10 mg of Resomer^®^ RG 502 H, Resomer^®^ RG 752H or Resomer^®^ RGP d5055 and 0.5 to 4 mg of sorafenib were dissolved in between 0.5 and 1.0 mL of acetone under magnetic stirring. A water phase with a volume of 2.0 to 4.0 mL was composed of an aqueous solution (0.5 to 2.0% *w/v*) of emulsifying agent (polysorbate 80, poly(methacrylic acid sodium salt), Triton X-100, or Pluronic^®^ F127), and added as a one-shot to the organic phase. Afterwards, the organic solvent was evaporated over a time period of 12 h at room temperature and 1 bar under constant stirring. The nanoparticles were centrifuged (Eppendorf 5424 R, Hamburg, Germany) at 37,565 *g* for 25 min, washed thrice and redispersed in an equal volume of purified water.

### 2.4. Preparation of Nanoparticles Using the Single Emulsion Technique

For the preparation of nanoparticles with the single emulsion–solvent evaporation method, the organic phase was prepared in two steps. An measure of 1 to 2 mg sorafenib was dissolved in 0.1 to 0.2 mL of acetone, and this solution was poured into a solution comprising 5 to 20 mg of Resomer^®^ RG 502 H, Resomer^®^ RG 752H, or Resomer^®^ RGP d5055 in 1 to 2 mL DCM. 

A volume of 4 to 8 mL of an aqueous solution of PVA (1 to 2% *w/v*) was added and sonicated using a Sonoplus HD2070, MS73 probe (Bandelin, Berlin, Germany) at an amplitude of 10% for 60 s. The organic solvent was evaporated over 2 h at atmospheric pressure and room temperature. The nanoparticles were purified as described above.

### 2.5. Preparation of Nanoparticles Using Double Emulsion–Solvent Evaporation Technique

The double emulsion–solvent evaporation technique was tested for the co-encapsulation of HSA into the sorafenib nanoparticles. The entrapped protein was further used for the covalent modification of the particle surface using the Gd-DTPA complex. In principle, the inner water phase was formed of 2.5 mg HSA in a volume of 0.1 mL of purified water. This solution was added to the organic phase, composed of 15 mg of Resomer^®^ RG 752H or Resomer^®^ RGP d5055 dissolved in 1.5 mL DCM, combined with 1.5 mg of sorafenib dissolved in 0.15 mL acetone. The first emulsification was performed by sonication using a Sonoplus HD2070, MS73 probe (Bandelin, Berlin, Germany) at an amplitude of 10% for 30 s. Afterwards, the water-in-oil emulsion was pipetted into 6.0 mL of an aqueous solution of PVA (1% (*w/v*)). A water-in-oil-in-water emulsion was formed by a second sonication step at an amplitude 15% for 45 s. The organic solvents were evaporated over a time period of 2 h under magnetic stirring at atmospheric pressure and room temperature. The nanoparticles were centrifuged at 37,565 *g* for 25 min (Eppendorf Centrifuge 5424 R), washed thrice and redispersed in 0.5 mL of phosphate buffer (pH 8), and, after gravimetric analysis, the suspensions were diluted to 20 mg∙mL^−1^ nanoparticle concentration.

### 2.6. Particle Morphology and Particle Size Analysis

The morphology of the nanospheres was investigated after centrifugation and redispersion in the described medium. The samples were examined using a FEI Talos F200XG2 high-resolution analytical microscope operated at 200 keV (Thermo Fischer Scientific, Waltham, MA, USA).

Additionally, the particle size and size distribution were determined using a Zetasizer Nano ZS (Malvern Instruments, Malvern, UK) equipped with a backscatter detector at an angle of 173°. The particles were characterized for their intensity mean diameter and polydispersity index (PDI). Dynamic light scattering (also known as photon correlation spectroscopy) is based on the Brownian motion of particles dispersed in a liquid. The particle diameter is calculated from the intensity fluctuations of light scattered at the particle surface. The Zetasizer Nano ZS uses a HeNe gas laser to generate a signal of these intensity fluctuations, from which the size is calculated by applying the Stokes–Einstein equation. Consequently, the resulting size distribution is an intensity distribution.

### 2.7. Storage Stability of Nanoparticle Formulations

To improve the physical stability of the colloidal dispersion during storage, the nanoparticles were freeze-dried, and their storage stability was investigated after 6 months of storage. A concentration of 3% (*w/v*) of two lyoprotectors (trehalose dihydrate or mannitol) was evaluated. The solid concentration was adjusted to 7 mg∙mL^−1^ of nanoparticles and the samples were put into a Christ Epsilon 2–7 freeze dryer (Martin Christ GmbH, Osterode am Harz, Germany). The lyophilisation was conducted in two drying steps. 

Initially, the temperature was decreased to −60 °C for 1 h to freeze the samples. Afterwards, primary drying was initiated by evacuating the chamber to 0.94 mbar. In parallel, temperature was raised to −30 °C over a time period of 150 min. The pressure was then reduced to 0.006 mbar and the temperature was increased to −10 °C over a time period of 60 min. These conditions were maintained for 35 h. The second drying was accomplished at a temperature of 10 °C for 60 min and at 20 °C for 10 h. The freeze dried samples were stored at 4 °C for a total duration of 6 months, and reconstituted in the same volume of purified water. The nanocomposite size and size distribution after redispersion were characterized by triplicated measurement.

### 2.8. Nanoparticle Yield and Encapsulation Efficiency

The nanoparticle yield was determined by microgravimetry. The drug loading and encapsulation efficiency were investigated dissolving 10 mg nanoparticles in 1 mL of DMSO. The solution was diluted using DMSO to be within the detectable linear calibration range (1–20 μg/L). The absorbance of the solutions was measured spectrophotometrically (Hitachi U-3000, Tokyo, Japan) at 285 nm. A concentration range of HSA solution in DMSO was prepared in order to correct the absorbance of HSA-loaded nanoparticles, also measured at 285 nm.

### 2.9. Biorelevant In Vitro Drug Release Test Using the Centrifugation Method

The biorelevant in vitro drug release test was conducted using the centrifugation method. In brief, 1.5 mg of the sorafenib-loaded nanocomposites was re-suspended in 1 mL of human blood plasma containing 0.03% sodium azide as a preservative. The nanoparticle formulations in release medium were filled into 2 mL tubes, and incubated at a temperature of 37 °C in a Thermomixer (Eppendorf, Hamburg, Germany) for 12 days at 700 rpm. At predetermined time points, 0.2 mL samples were collected. After each sampling time point the medium was replenished. The nanoparticles were separated from the plasma by centrifugation (Eppendorf Centrifuge 5424 R, 20 min at 37,565 *g*). The concentration of free sorafenib was determined using direct quantification of the drug remaining in the particle system. For this purpose, the nanoparticle pellets were dissolved in DMSO and the drug amount was detected spectrophotometrically.

### 2.10. Surface Modification Using Human Serum Albumin and Polyethyleneimine

The nanoparticle dispersions prepared by single emulsion technique were centrifuged and redispersed in phosphate buffer (pH 8), resulting in a nanoparticle concentration of 20 mg∙mL^−1^. To increase the number of free amino groups on the particle surface, HSA or PEI were covalently bound to the nanoparticle surface. A 50-fold molar excess of EDC and the same excess of NHS, both calculated to the molar polymer concentration, were dissolved in 0.5 mL phosphate buffer (pH 8) and incubated for 60 min, centrifuged and washed three times, and redispersed in 1.5 mL phosphate buffer (pH 8). 

The obtained carbodiimide-activated nanoparticle dispersion was added into 0.5 mL phosphate buffer (pH 8) solution containing equimolar amounts of HSA or PEI, and shaken overnight at 20 °C in an Eppendorf Thermomixer (Hamburg, Germany) at 700 rpm. Afterwards, the nanoparticles were dialysed for 2 h against 400 mL of purified water, using a 100 kDa membrane to remove residues of the dissolved polymers HSA or PEI, respectively. The dialysis step was repeated with purified water.

### 2.11. Conjugation of Nanoparticles Using the Gd-DTPA Complex

The Gd-DTPA complex was covalently attached to either HSA or PEI after activation using the carbodiimide. In order to activate the carboxyl groups of the gadolinium complex, a 10-fold molar excess of Gd-DTPA (calculated on the amount of the Resomer^®^) was dissolved in 1 mL of buffer (pH 8) and combined with a 5-fold molar excess of EDC and a 5-fold molar excess of NHS (calculated on the amount of Gd-DTPA). The resulting solution was incubated for 50 min and added to the purified nanoparticle suspension overnight. The preparation was purified by dialysis twice, using a membrane with a MWCO of 3.5 kDa and 500 mL of purified water for 2 h. The dialysed nanoparticle suspension was centrifuged and redispersed in 1.5 mL phosphate buffer.

### 2.12. Quantification of Human Serum Albumin Using the Micro Bicinchoninic Acid Method

The crosslinked or co-encapsulated HSA content of the nanocomposites was determined by the µBCA method after centrifuging 0.1 mL nanoparticle dispersion and removing the supernatant, while the pellet was dissolved in 0.5 mL DMSO. This solution was diluted 10-fold with purified water, and incubated for 1 h at 60 °C with the same volume of freshly prepared µBCA reagents mixture. DMSO was added to the calibrating HSA solution with the same ratio. After cooling the colored mixtures to room temperature for 20 min, their absorbance was evaluated by spectrophotometry at 562 nm (Hitachi U-3000, Tokyo, Japan).

### 2.13. Quantification of Gadolinium Using Inductively Coupled Plasma Optical Emission Spectroscopy

The Gd concentration was measured using a Spectro Genesis ICP-OES (Kleve, Germany) simultaneous spectrometer with axial plasma observation. Multielemental standards (Merck, standard solutions for ICP, Darmstadt, Germany) were used for calibration. The limits of detection of the element were calculated according to Equation (1):

(1) $$Limit\;of\;detection=\;Background\;signal+3\times SD_{Background}\times f_{dilution}$$

The purified nanoparticles were dissolved in 5 M hydrochloric acid and diluted to the desired calibration range.

### 2.14. In Vitro Investigation of Diagnostic Features by Magnetic Resonance Imaging

In vitro MRI was carried out using box analysis to compare the contrast achieved with the nanoparticle suspension with that of Magnevist^®^ (Gd-DTPA complex with meglumine) solution. A calibration was achieved by diluting Magnevist^®^ to 0.01–2.5 mg∙mL^−1^ concentration. MR imaging was performed on a 3.0-T scanner (Siemens Magnetom Trio, Siemens Medical Solutions, Erlangen, Germany). The measurement conditions were T1-weighted 3D gradient echo sequences (fast low-angle shot) with the following parameters: TE (echo time) = 3.31 ms, TR (repetition time) = 8.67 ms, field of view = 100 × 78 mm, matrix acquisition = 640 × 480, slice thickness = 0.3 mm, flip angle = 16°, fat suppression = fat saturated, and bandwidth = 180 Hz/Px.

### 2.15. Labeling of Nanoparticles with a Fluorescent Dye

A volume of 1.0 mL of the nanoparticle suspension (12 mg∙mL^−1^) in phosphate buffer (pH 8) was added to 0.1 mL phosphate buffer (pH 8) containing a 25-fold molar excess of EDC and NHS (calculated on the amount of Resomer^®^) and incubated for 60 min, centrifuged, and washed, and redispersed in 1.0 mL phosphate buffer (pH 8). The obtained carbodiimide activated nanoparticle dispersion was given to 0.1 mL phosphate buffer (pH 8) solution containing 1 mg∙mL^−1^ Cyanine5 amine fluorescent (Cy5) dye, and shaken for 1 h at 20 °C in an Eppendorf Thermomixer (Hamburg, Germany) at 700 rpm. The nanoparticle dispersion was then centrifuged in an Eppendorf Centrifuge 5424 R (Hamburg, Germany) at 37,565 *g* for 25 min, washed three times, and redispersed in phosphate-buffered saline to a nanoparticle concentration of 10 mg∙mL^−1^.

### 2.16. In Vitro Cellular Uptake and Cytotoxicity

Cellular uptake of the nanoparticles into the HepG2 cells was evaluated using flow cytometry. The cells were cultured in 24-well plates at a cell density of 2 × 10^5^ cells per well at 37 °C and 5% CO_2_ for 24 h. After cultivation, 100 μg nanoparticles/well (10 mg/mL nanoparticle suspension was diluted to 100 μg/mL) was pipetted to the cells and incubated for 24 h. The cells grown without nanoparticles were used as control. The cells were washed in phosphate-buffered saline (PBS), trypsinised, and redispersed in PBS containing 2% (*m/v*) of bovine serum albumin. Flow cytometry was performed on a Cytoflex S cytometer (Beckman Coulter, Brea, CA, USA).

To further analyze cellular uptake and intracellular localization, fluorescence microscopy of HepG2 cells plated on cover slides and incubated with either Cy5-labeled PLGA or PEG-PLGA NP for 4 h and 24 h was employed. Membrane staining was performed by using Alexa488 concanavalin A (2.5 µg/mL; Thermo Fisher Scientific, Schwerte, Germany) and nuclei were counterstained with 4’,6-diamidino-2-phenyl-indole (DAPI) solution (Merck, Darmstadt, Germany). Finally, slides were mounted with Vectashield mounting medium (Biozol, Eching, Germany) and images were obtained using an AxioImager Z1 microscope and Axiovision 4.6 software (Carl Zeiss, Jena, Germany).

The in vitro cytotoxicity in HepG2 cells was determined using 3-(4,5-dimethylthiazol-2-yl)-2,5-diphenyltetrazolium bromide (MTT) assay. Cells were seeded (50,000 cells/well) in 96-well plates. At 24 h pre-incubation, the media were replaced with 100 μL of fresh Dulbecco’s Modified Eagle’s Medium (DMEM) containing 10% FBS and sorafenib-loaded nanoparticles. Three different sorafenib concentrations (6.25 μg/mL, 12.5 μg/mL, and 25 μg/mL) were used, while the control samples contained the same amounts of free sorafenib in DMSO solution. After 24 h of incubation, a volume of 10 μL per well of MTT solution (5 mg MTT/mL) was added, followed by further incubation for 2 h. The supernatant was removed, and 0.2 mL MTT lysis solution was added into each well. The absorbance of cell suspension was determined at 595 nm using a spectrophotometer (EnVision 2104 Multilabel Reader, Perkin Elmer, Waltham, MA, USA). The percentage of viable cells was calculated by comparing the absorbance of treated cells against the untreated cells (negative control). The DMSO solution and the blank nanoparticle suspensions served as positive controls. The data are presented as mean and standard deviation with five replicates.

### 2.17. Statistics

All data are expressed as the mean value ± standard deviation (SD), which were calculated and plotted using Microsoft Excel (Microsoft, Redmond, WA, USA) and SigmaPlot 11.0 (Systat Software GmbH, Erkrath, Germany), respectively. All nanoparticle formulations were produced as three batches (*n* = 3).

## 3. Results and Discussion

In recent years, a variety of preparation methods have been evaluated for the synthesis of nanocarrier devices. The current study produced advanced theranostic drug carriers and compared the impact of manufacturing technology and surface modification on their physicochemical properties and in vitro features.

**Figure 1:**
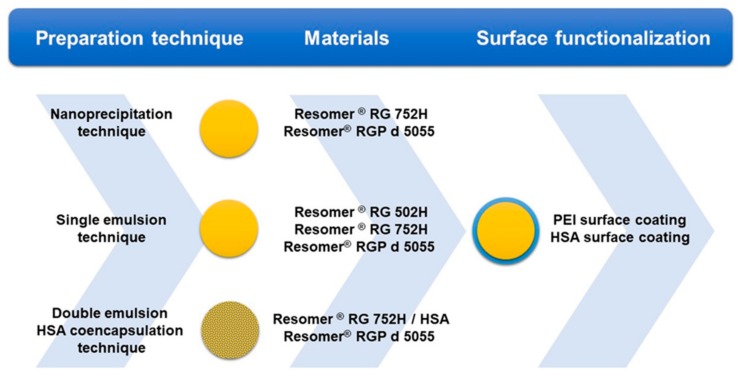
Scheme of the nanoparticle preparations with the preparation technique, materials used, and surface functionalization (from the left to the right). Each preparation technique was evaluated with the presented materials. Surface coating was undertaken for preparations manufactured by single emulsion technique only.

Initially, a particle size between 100 and 300 nm [17], a zeta potential of more than −15 mV [18,19], and a high drug load and particle yield were identified as key criteria for formulation development. Due to the enhanced permeability and retention (EPR) effect, these nanoparticles may be capable of targeting tumor tissues [17]. While smaller nanoparticles can be rapidly excreted by the kidneys, larger colloids with a size of more than 300 nm are quickly recognized by the macrophages of the reticuloendothelial system [11]. Among other aspects, the encapsulation efficiency and particle size play an important role in nanocarrier delivery. Selecting biodegradable polymers of the Resomer^®^ type, three different techniques for the encapsulation of sorafenib were compared (Figure 1).

### 3.1. Manufacture of Sorafenib-Loaded Core Particles Using Nanoprecipitation Technique

The manufacture of sorafenib-loaded nanoparticles by nanoprecipitation using Triton X-100 or Pluronic^®^ F127 stabilizers in aqueous solution resulted in a pronounced aggregation for all three polymers. Similar observations have been made at the medium scale, suggesting poor ‘scalability’ during the later stages of production [8]. Changing the stabilizer to polysorbate 80, similar aggregation occurred with Resomer^®^ RG 502H and Resomer^®^ RG 752H, respectively.

**Figure 2:**
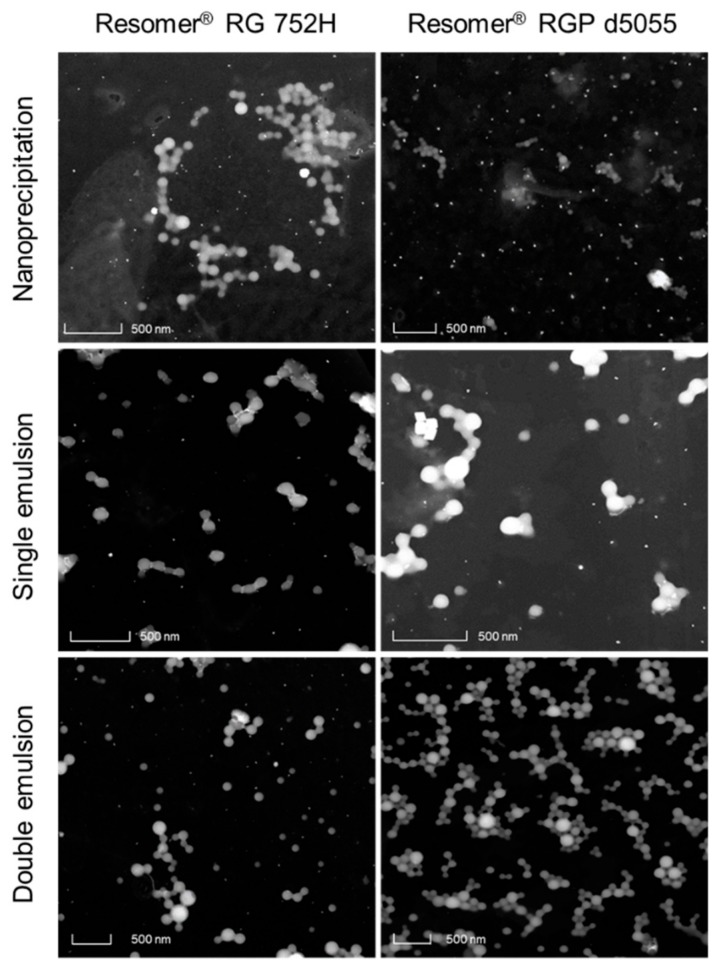
Scanning/transmission electron micrographs of nanoparticles comprising sorafenib in a Resomer^®^ RG 752H (**upper left**) or Resomer^®^ RGP d5055 (**upper right**) matrix prepared by nanoprecipitation, in a Resomer^®^ RG 752H (**middle left**) and Resomer^®^, RGP d5055 (**middle right**) matrix prepared by single emulsion–solvent evaporation technique using polyvinyl alcohol (PVA) as a stabilizer, or in a Resomer^®^ RG 752H-HAS (**lower left**) and Resomer^®^, RGP d5055-HSA (**lower right**) matrix by double emulsion–solvent evaporation technique using PVA as a stabilizer.

The use of Resomer^®^ RGP d5055 (and polysorbate 80) resulted in nanoparticles within the desirable size range (153 ± 14 nm, Figure 2, upper micrographs). However, the PDI of more than 0.27, an encapsulation efficiency below 20%, and a particle yield between 20 and 40% were the major disadvantages of this formulation design. A lower density of the particle system due to the hydrophilic side chains of the polymer is the most likely explanation. 

**Figure 3:**
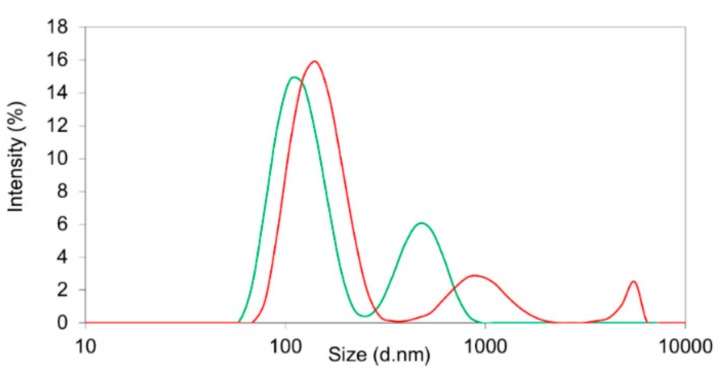
Size distribution by intensity of nanocomposites containing sorafenib in Resomer^®^ RG 502H (green line) and Resomer^®^ RGP d5055 (red line) matrix nanoparticles prepared by nanoprecipitation, using PVA as an emulsifier.

In comparison, the nanoprecipitation technique using PVA in combination with Resomer^®^ RG 502H or Resomer^®^ RGP d5055 led to particle systems broadly distributed in size, as indicated by elevated polydispersity indices ranging between 0.27 and 0.46. This was also confirmed by the intensity distributions, exhibiting a second and third fraction of larger particles in the micrometer range (Figure 3). The zeta potential of sorafenib-loaded Resomer^®^ RG 502H or Resomer^®^ RGP d5055 nanocomposites by nanoprecipitation and PVA emulsifier was found to be 13.2 ± 0.7 mV and 12.1 ± 1.2 mV, which also explains the poor stability resulting in a pronounced aggregation. Under similar conditions, crystallization of the drug accompanied by an increased PDI between 0.21 and 0.35 was reported by Lin et al. [18]. When applying the nanoprecipitation technique, a particle size in the desired range (196 ± 10 nm) as well as a reduced PDI of 0.21 ± 0.03, was achieved when using a polymer concentration of 10 mg∙mL^−1^ of Resomer^®^ RG 752H and 1 mg∙mL^−1^ of sorafenib in the acetone phase.

Considering the difficulties in nanoparticle preparation when using the nanoprecipitation method, later efforts were focused on nanoparticle manufacture by single and double emulsion techniques.

### 3.2. Manufacture of Sorafenib-Loaded Core Particles by Single Emulsion Technique

**Figure 4:**
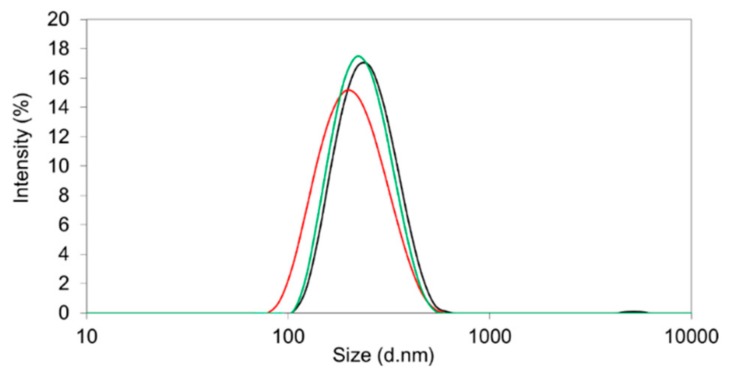
Size distribution by intensity of sorafenib-containing nanoparticles prepared from Resomer^®^ RG 502H (black line) and Resomer^®^ RG 752H (red line) polylactic-*co*-glycolic acid (PLGA) polymers as well as Resomer^®^ RGP d5055 (green line) by single emulsion method.

For the preparation of nanoparticles utilizing the single emulsion method, the hydrophilic phase was composed of a 1% or 2% (*w/v*) aqueous solution of PVA in purified water (Table 1). A polymer concentration of 10 mg∙mL^−1^, an initial drug amount of 10% (*m/m*), and a water-to-dichloromethane (DCM) ratio of 4:1 led to acceptable properties for each of the three polymers (Table 1). The morphology of the nanoparticles was tested by scanning/transmission electron microscopy. They were of spherical shape and within the expected size range (Figure 4).

With an encapsulation efficiency ranging between 70.4 and 78.8% (Table 1) and a monomodal size distribution (Figure 4), the emulsion method was superior compared to the nanoprecipitation technique. The drug loading (8.9–12.0% *m/m*) almost reached the initial drug ratio (10% *m/m*) (Table 1). Further increase of the drug amount from 10% (*m/m*) to 20% (*m/m*) resulted in elevated PDI values due to the formation of larger aggregates. 

**Figure 5:**
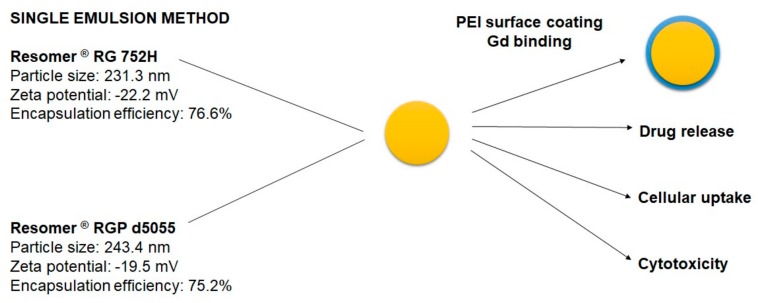
Selected formulations for surface modification and further tests.

The best outcomes were achieved by using Resomer^®^ RG 752H or Resomer^®^ RGP d5055 as matrices (Figure 2, middle micrographs). Resomer^®^ RG 752H resulted in a particle yield of 73.7%, an encapsulation efficiency of 76.6%, and a drug load of 11.2% (Figure 5). For Resomer^®^ RGP d5055, similar promising results were achieved. A particle yield of 76.1%, an encapsulation efficiency of 75.2%, and a drug load of 8.9% were obtained using a 2% *m/v* emulsifier concentration. These nanocomposites were selected for further surface modification and cellular uptake and cytotoxicity studies (Figure 5). The zeta potential indicated high colloidal stability, which was also confirmed during further processing. It is likely that the surface charge resulted from the high number of carboxyl groups present in the PLGA polymers. However, there were no major differences between the zeta potential values of the employed polymers even when the nanocarriers were manufactured from the PEGylated derivative. This indicates that PEGylation did not significantly reduce the number of the carboxyl groups. These nanoparticle preparations were further processed and evaluated for their release behavior.

### 3.3. Co-Encapsulation of HSA Using Double Emulsion Method or Surface Modification

To covalently bind the Gd-DTPA complex to the particle surface, amino groups were introduced into the polymeric matrices. In a first approach, HSA was co-encapsulated into the nanoparticles using the double emulsion–solvent evaporation method. Although there was no significant difference between the diameters of nanoparticles manufactured from Resomer RG 752H (mean diameter 210.6 nm, PDI 0.113) and Resomer^®^, RGP d5055 polymers (mean particle diameter 210.3 nm, PDI 0.099), incorporation of the protein resulted in smaller particle sizes than the single emulsion method. The detected size range was confirmed by electron microscopy (Figure 2, lower micrographs). All nanocomposites were of spherical shape (Figure 3). The particle yield was much lower compared to the methods described earlier. A particle yield of 50.4 ± 1.0% for Resomer^®^ RG 752H and 49.0 ± 5.3% for Resomer^®^ RGP d5055 was achieved. A similar amount of HSA was incorporated into both polymers (Resomer^®^ RG 752H 10.6 ± 1.1%, Resomer^®^ RGP d5055, 9.4 ± 1.2%). The formation of smaller particles with reduced density could be responsible for the decreased particle yield. 

However, the incorporation method resulted in a high protein loading and an increased number of functional groups available for the EDC reaction. In comparison, an amount of 1.0 ± 0.3% HSA for Resomer^®^ RG 752H and 1.9 ± 0.5% HSA for Resomer^®^ RGP d5055 was bound to the particles using the surface coating technique.

### 3.4. Surface Modification with Gadolinium

The Gd-DTPA complex was conjugated to the amino groups of the HSA molecules after incorporation or covalent binding of the protein to Resomer^®^ RG 752H and Resomer^®^ RGP d5055 nanoparticles, respectively. As expected, the modification of nanocomposites with Gd-DTPA was limited by the availability of functional groups on the particle surface. 

The nanoparticles modified on their surface with HSA were characterized by poor protein binding, with an amount of approximately 1.5 mg gadolinium per g Resomer^®^ RG 752H, and 1.4 mg gadolinium per g Resomer^®^ RGP d5055. Nanoparticles comprising HSA as part of their matrix structure exhibited a much higher binding of 2.3 mg gadolinium per g Resomer^®^ RG 752H and 3.2 mg gadolinium per g Resomer^®^ RGP d5055. 

Nevertheless, further increase of the gadolinium content was achieved using a method described previously [20] with some modification. The binding of PEI to the surface resulted in a significant increase of the gadolinium content, with 15.7 mg gadolinium per g Resomer^®^ RG 752H and 10.7 mg gadolinium per g Resomer^®^ RGP d5055. 

### 3.5. Evaluation of Contrast Signal Using In Vitro MRI

The MRI properties of theranostic nanocarriers were investigated in vitro. Magnevist^®^ aqueous solutions were used as a reference. There was a linear correlation (*R*^2^ = 0.9724) between the MRI signal and the gadolinium content in the range between 0.02 and 0.3 mg∙mL^−1^ (with MRI intensities of 470–670 at 0.02 mg∙mL^−1^, and 1520–1750 at 0.3 mg∙mL^−1^). For nanoparticles exhibiting the highest content of gadolinium, the theoretical particle concentration (calculated from a maximum injectable particle concentration of 10 mg∙mL^−1^ and the gadolinium load) fell into this calibration range. The signal intensities measured for the T1-weighed MRI were in good accordance with the inductively coupled plasma optical emission spectroscopy (ICP-OES). Earlier studies reported a loading of 4.7 to 8.5 mg gadolinium per g HSA nanoparticles after covalent binding of DTPA. This amount was found to be considerably high for in vivo MRI studies [5].

### 3.6. Storage Stability of Theranostic Nanocomposite Formulations

**Figure 6:**
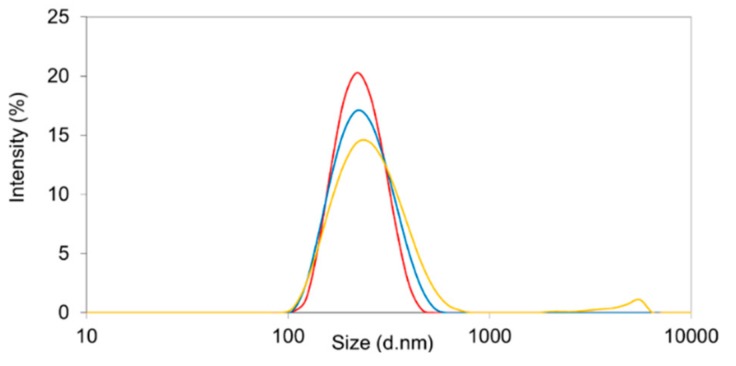
Size distribution by intensity of Resomer^®^ RGP d5055 nanoparticles after preparation (red line), and following reconstitution after freeze-drying in the presence of 3% of trehalose (blue line) and 3% of mannitol (yellow line).

To freeze dry Resomer^®^ RG 752H and Resomer^®^ RGP d5055 nanoparticles manufactured by the single emulsion method, a concentration of 3% (*w/v*) of the lyoprotectors sucrose, trehalose, and mannitol has been previously applied [21]. The sorafenib-loaded nanoparticles with a drug load of approximately 10% were freeze dried in the presence of 3% (*w/v*) of trehalose or mannitol. The nanospheres remained stable over the time of storage. An increase in the PDI values was observed for formulations freeze-dried with mannitol. On this basis, trehalose at a concentration of 3% was identified to be the optimal lyoprotector for the developed particle system (Figure 6).

### 3.7. Evaluation of In Vitro Drug Release of Sorafenib from Theranostic Nanocomposites

For nanoparticles comprising polymers from the Resomer^®^ family, a biphasic release pattern has been previously reported [22]. Preliminary investigations indicated a less pronounced burst release for Resomer^®^ RG 752H compared to Resomer^®^ RG 502H. After the initial release phase, a sustained release behavior was observed.

**Figure 7:**
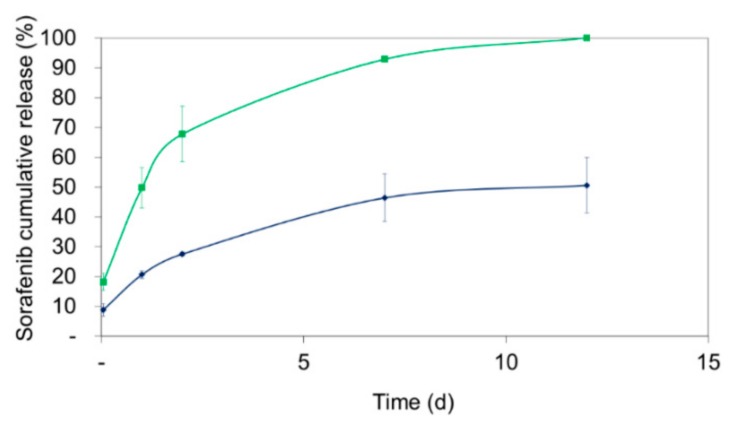
Sorafenib release from Resomer^®^ RG 752H (green line) and Resomer^®^ RGP d5055 -sorafenib (PEG-PLGA-SFB) nanocomposites (blue line) in human blood plasma. Data are presented as mean ± SD from three independent samples for each concentration.

Consequently, the drug release of sorafenib-loaded Resomer^®^RGP d5055 and Resomer^®^ RG 752H particles was investigated over a time period of 12 days in human blood plasma. Both of the nanocomposites were prepared under optimized conditions, that is, using the single emulsion method with an encapsulating polymer concentration of 10 mg∙mL^−1^, an initial drug amount of 10% (*m/m*), a water-to-DCM ratio of 4:1, and 2% (*w/v*) of PVA. The initial burst release was higher with nanoparticles manufactured from Resomer^®^ RG 752H polymer (18.2 ± 2.9%) compared to Resomer^®^RGP d5055 copolymer, from which 8.8 ± 2.1% of the drug was released during the first hour. Afterwards, a continuous release was observed for both composites (Figure 7). Surprisingly, the release from Resomer^®^RGP d5055 was substantially slower, reaching a plateau at 50.6 ± 9.2%. A specific interaction of sorafenib with the hydrophilic side chain of the block copolymer could be responsible for this behavior.

### 3.8. Cellular Uptake and Cytotoxicity

**Figure 8:**
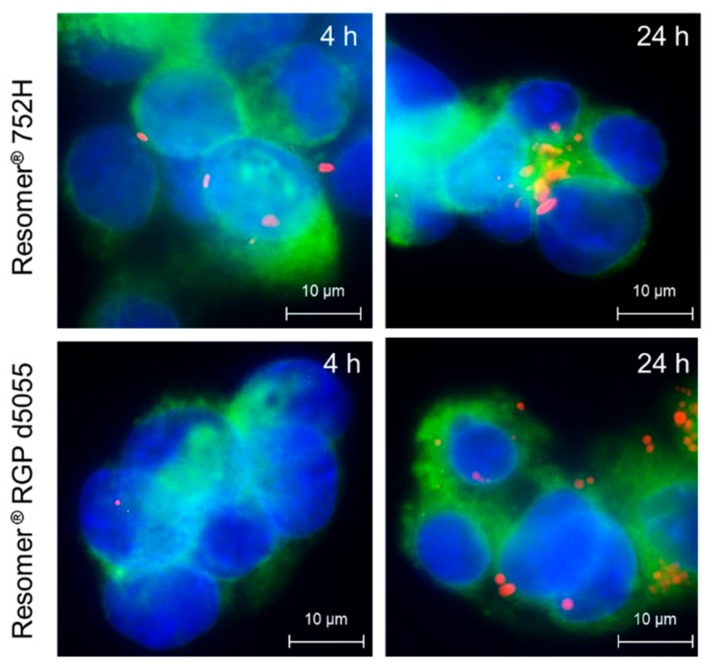
Cellular uptake of either Cy5-conjugated Resomer^®^ 752H or Resomer^®^ RGP d5055 nanoparticles (red). Membrane staining was performed by using Alexa488 concanavalin A (green). Nuclei were stained with 4′,6-diamidino-2-phenylindole (DAPIblue).

For the cellular uptake and cytotoxicity studies, sorafenib-loaded Resomer^®^RGP d5055 and Resomer^®^ RG 752H nanocomposites were prepared by the single emulsion method at a polymer concentration of 10 mg∙mL^−1^, an initial drug amount of 10% (*m/m*), a water-to-DCM ratio of 4:1, and 2% (*w/v*) of PVA. The internalization rates of sorafenib-containing nanoparticles in HepG2 cells were quantified by flow cytometry. Additionally, fluorescence microscopy was conducted to confirm the localization of the particles inside the cells (Figure 8). 

**Figure 9:**
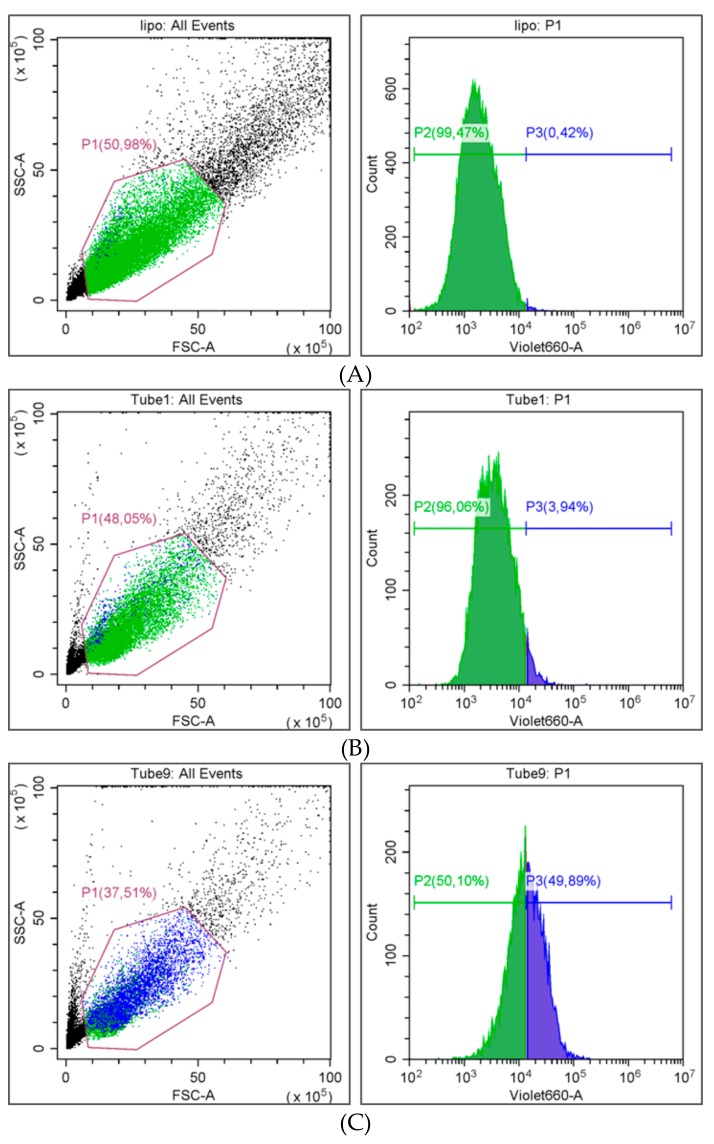
Flow cytometry diagrams of untreated cell control (**A**), sorafenib-loaded Resomer^®^RGP d5055 (**B**), and Resomer^®^ RG 752H nanocomposites (**C**).

As expected, the uptake of drug-loaded nanoparticles prepared by using the PEGylated polymer was significantly lower (3.9 ± 3.3%, Figure 9B) than for Resomer^®^ RG 752H nanoparticles (49.9 ± 4.9%, Figure 9C). The biocompatibility of nanomedicines is an important feature with regards to clinical success and commercialization. 

**Figure 10:**
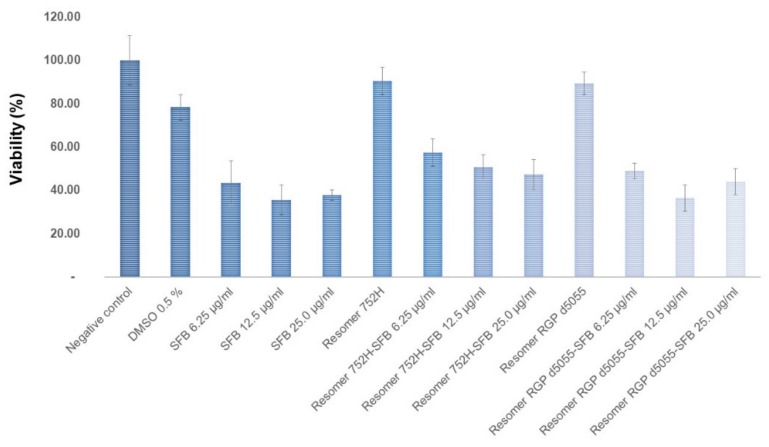
Viability of HepG2 cells treated with different concentrations of sorafenib (SFB) or Resomer^®^ RG 752H or Resomer^®^ RGP d5055 nanoparticles, and that of untreated cells (negative control).

The cytotoxicity of the unloaded and the drug-loaded nanoparticles was investigated in HepG2 cells. Solutions of the drug in dimethyl sulfoxide (DMSO), the organic solvent alone, and an untreated negative control were used as references. As shown in Figure 10, the viability of the cells remained at approximately 90% when exposed to 100 μg/well of both types of unloaded nanoparticles (50,000 cells/well). The non-toxic features of Resomer^®^ polymers have been verified previously [4].

As expected, sorafenib exhibited a concentration-dependent cytotoxicity in the HepG2 cells. Similar concentrations of sorafenib resulted in higher cytotoxicity for the free drug compared to the particle formulations (Figure 10), which could have been due to the slow release of the drug from the particle matrix. 

## 4. Conclusions

Theranostic nanocomposites comprising PLGA or PEG-PLGA were loaded with the anti-tumor drug sorafenib and modified on their surface with the contrast agent Gd-DTPA. The single emulsion technique was found to be the most appropriate method for the effective preparation of monodisperse nanocomposites. These nanospheres exhibited superior properties compared to the particle systems described in the literature. Finally, the top-down manufacture combined with a modification of the particle surface using PEI and Gd-DTPA resulted in a strong MRI signal, optimal stability features, and a sustained release profile. On this basis, further investigations will focus on the in vivo performance of these nanocarriers.

## Figures and Tables

**Table 1:** Properties of Resomer–sorafenib nanoparticles as a function of the encapsulating polymer and emulsifier (PVA) concentration.

Material	Resomer^®^ RG 502H	Resomer^®^ RG 752H	Resomer^®^ RGP d5055	Resomer^®^ RG 502H	Resomer^®^ RG 752H	Resomer^®^ RGP d5055
PVA (% *w/v*)	1	1	1	2	2	2
Mean size by intensity (nm)	235 ± 2.0	227.7 ± 3.3	228.3 ± 8.0	231.4 ± 15.6	231.3 ± 30.1	243.4 ± 40.4
PDI	0.14 ± 0.04	0.18 ± 0.01	0.12 ± 0.02	0.15 ± 0.06	0.19 ± 0.04	0.15 ± 0.14
Encapsulation efficiency (%)	70.4 ± 3.5	76.2 ± 2.1	76.7 ± 2.6	78.8 ± 4.4	76.6 ± 2.7	75.2 ± 6.7
Drug loading (%)	9.0 ± 0.5	10.2 ± 0.3	10.0 ± 0.4	12.0 ± 0.2	11.2 ± 0.1	8.9 ± 0.4
Zeta potential (mV)	−21.3 ± 2.4	−22.4 ± 2.4	−19.7 ± 1.1	−19.8 ± 3.3	−22.2 ± 1.8	−19.5 ± 1.6
